# Mechanisms and Therapeutic Potential of Sodium–Glucose Cotransporter 2 Inhibitors in Heart Failure

**DOI:** 10.31083/RCM45833

**Published:** 2026-04-20

**Authors:** Zuyuan Huang, Guoxing Ling, Chen Fang, Zimin Wu, Shigao Ye, Chuanliang Zhang, Cheng Luo, Baoshi Zheng

**Affiliations:** ^1^Department of Cardiovascular Surgery, The First Affiliated Hospital of Guangxi Medical University, 530021 Nanning, Guangxi, China

**Keywords:** sodium–glucose cotransporter 2 inhibitors, heart failure, mechanisms of action, clinical therapy

## Abstract

Heart failure (HF) is a major global cause of hospitalization and mortality, representing a complex clinical syndrome with significant unmet therapeutic needs. Sodium–glucose cotransporter 2 inhibitors (SGLT2is), originally developed for glycemic control, have recently demonstrated remarkable efficacy in the management of HF. This review comprehensively examines the mechanisms of action and therapeutic potential of SGLT2is in HF, with a focus on their multifaceted effects on hemodynamics, cardiac metabolism, inflammatory responses, oxidative stress, and neuroendocrine activation. In addition, clinical trial outcomes and safety profiles of SGLT2is in HF with reduced ejection fraction (HFrEF), HF with preserved ejection fraction (HFpEF), and HF with mid-range ejection fraction (HFmrEF) are thoroughly evaluated. Finally, this article discusses future research directions and clinical application prospects, aiming to provide novel insights and strategies for treating HF.

## 1. Introduction

Heart failure (HF) represents the terminal stage of various cardiovascular diseases, affecting over 64 million individuals worldwide, with its prevalence continuing to rise due to aging populations and the increasing incidence of metabolic disorders [1]. Despite advances in pharmacologic and device-based therapies—such as renin–angiotensin system inhibitors, β-blockers, and mineralocorticoid receptor antagonists (MRAs)—HF remains associated with high hospitalization and mortality rates, underscoring the need for novel and more effective treatments [2, 3, 4]. However, their limitations, including suboptimal efficacy and potential adverse effects with long-term use, underscore the need for novel therapeutic approaches [5].

Sodium–glucose cotransporter 2 inhibitors (SGLT2is), initially developed as antidiabetic agents that block renal glucose reabsorption, have emerged as a major therapeutic breakthrough in HF management [6]. Beyond their glucose-lowering effect, large-scale clinical trials and meta-analyses have demonstrated that SGLT2is significantly reduce cardiovascular death and HF hospitalizations and provide renal protection across diverse patient populations, including those without diabetes [6, 7, 8].

Despite the promising therapeutic potential of SGLT2is in HF, the complexity and heterogeneity of their mechanisms of action warrant further investigation [9]. This review aims to comprehensively explore the mechanisms and therapeutic potential of SGLT2is in HF, with an in-depth analysis of their multifaceted effects on hemodynamics, cardiac metabolism, inflammatory responses, oxidative stress, and neuroendocrine activation. Furthermore, we systematically evaluate clinical trial outcomes of SGLT2is in HF with reduced ejection fraction (HFrEF), HF with preserved ejection fraction (HFpEF), and HF with mid-range ejection fraction (HFmrEF), along with a detailed discussion of their safety profile in clinical practice [10]. By elucidating the multi-target effects of SGLT2is, this review seeks to provide more precise and effective therapeutic strategies for HF patients, ultimately improving their prognosis and alleviating the global healthcare burden.

## 2. Overview of SGLT2is

**Fig. 1.**
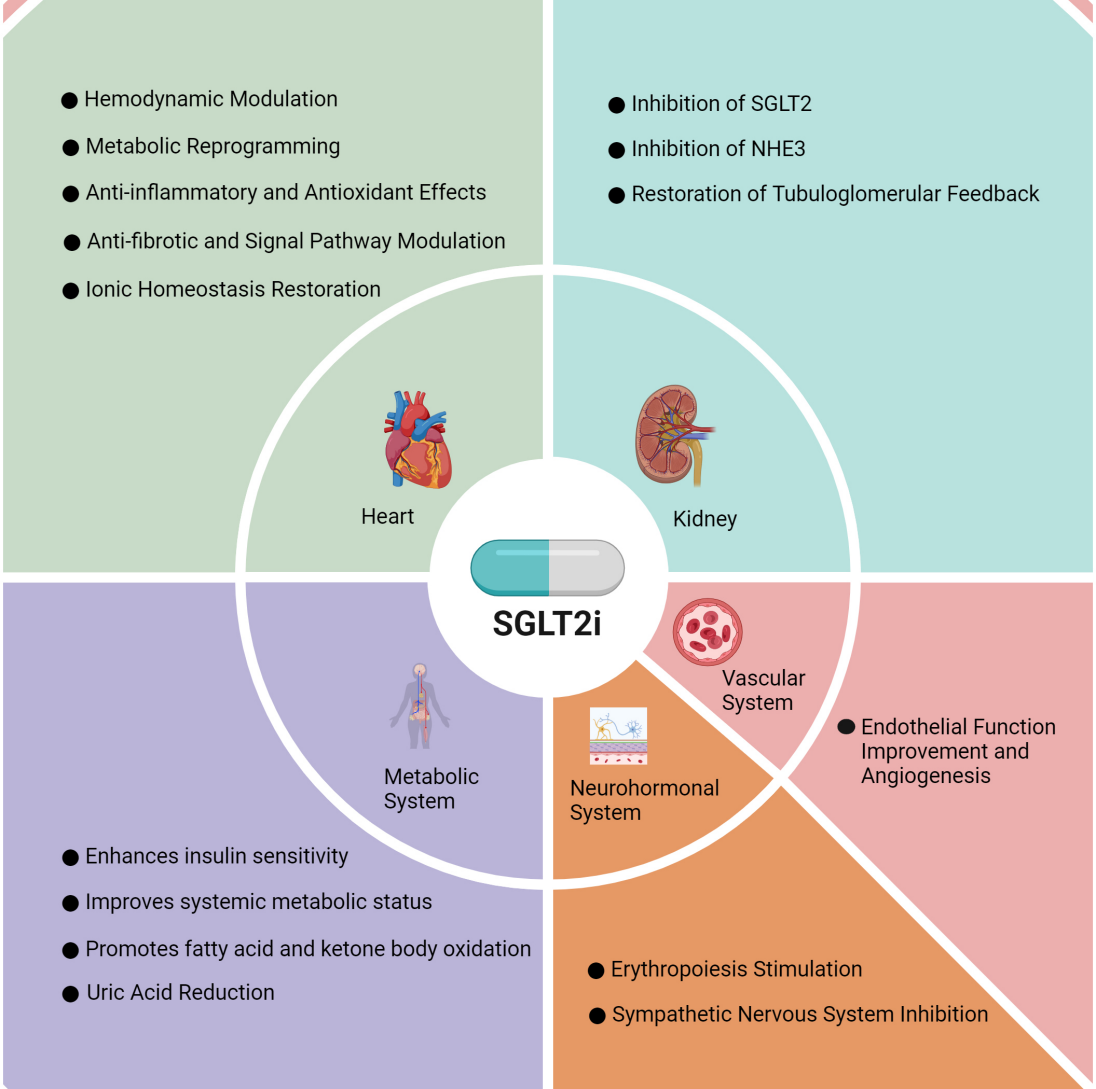
**Therapeutic mechanisms of SGLT2is in HF**. SGLT2is exert distinct therapeutic effects in the heart, kidney, metabolic system, neurohormonal system, and vascular system. NHE3, sodium–hydrogen exchanger 3; SGLT2is, sodium–glucose cotransporter 2 inhibitors; HF, heart failure. Fig. 1 was drawn using BioRender.

SGLT2is represent a novel class of oral antihyperglycemic agents that have received significant attention in the medical field due to their unique mechanism of action and broad clinical applications [11]. SGLT2is primarily function by inhibiting the activity of SGLT2 in the proximal renal tubules, thereby reducing glucose reabsorption and promoting urinary glucose excretion, which effectively lowers blood glucose levels [6] (Fig. 1). Beyond glycemic control, SGLT2is exert beneficial hemodynamic effects by increasing urinary sodium excretion and inducing osmotic diuresis, which alleviates cardiac preload, reduces blood pressure, and preserves cardiac function [12]. In addition, SGLT2is inhibit sodium–glucose cotransporter 1 (SGLT1) in cardiomyocytes, mitigating intracellular sodium overload, calcium overload, and oxidative stress, thereby improving ventricular remodeling and protecting myocardial tissue [13]. SGLT2is also modulate adipose tissue metabolism and inflammation, attenuate myocardial fibrosis, and enhance cardiac metabolic remodeling [13].

SGLT2is have a wide range of clinical applications, including use in adult patients with type 2 diabetes mellitus (T2DM) who have inadequate glycemic control with oral antihyperglycemic agents or basal insulin therapy, as well as in T2DM patients with comorbid cardiovascular disease, chronic kidney disease, or HF [14]. Commonly prescribed SGLT2is include dapagliflozin, empagliflozin, canagliflozin, and ertugliflozin. Numerous clinical trials have demonstrated that SGLT2is significantly reduce the composite endpoint of cardiovascular mortality and HF-related hospitalization in patients with both HFrEF and HFpEF [15, 16, 17]. SGLT2is also confer additional benefits, such as weight reduction, improved insulin sensitivity, decreased uric acid levels, and modest lowering of blood pressure [18]. In major heart failure trials, mean body weight reductions ranged between 1.5 and 2.5 kg within the first 6 months of therapy, and remained stable [19]. In Dapagliflozin And Prevention of Adverse-outcomes in Heart Failure (DAPA-HF), dapagliflozin led to an average weight loss of 1.8 kg compared with placebo at 8 months, while EMPEROR-Reduced and EMPEROR-Preserved trials reported similar findings [20]. Although SGLT2is are associated with an increased risk of urogenital infections and ketoacidosis, they are generally well tolerated in clinical practice, and most adverse effects are easily manageable [21]. Owing to their multifaceted mechanisms of action, SGLT2is have demonstrated remarkable efficacy and broad therapeutic potential in the management of T2DM and HF, offering new hope and expanded treatment options for patients.

## 3. Mechanisms of Action of SGLT2is in HF

### 3.1 Improving Hemodynamics and Reducing Interstitial Edema

**Fig. 2.**
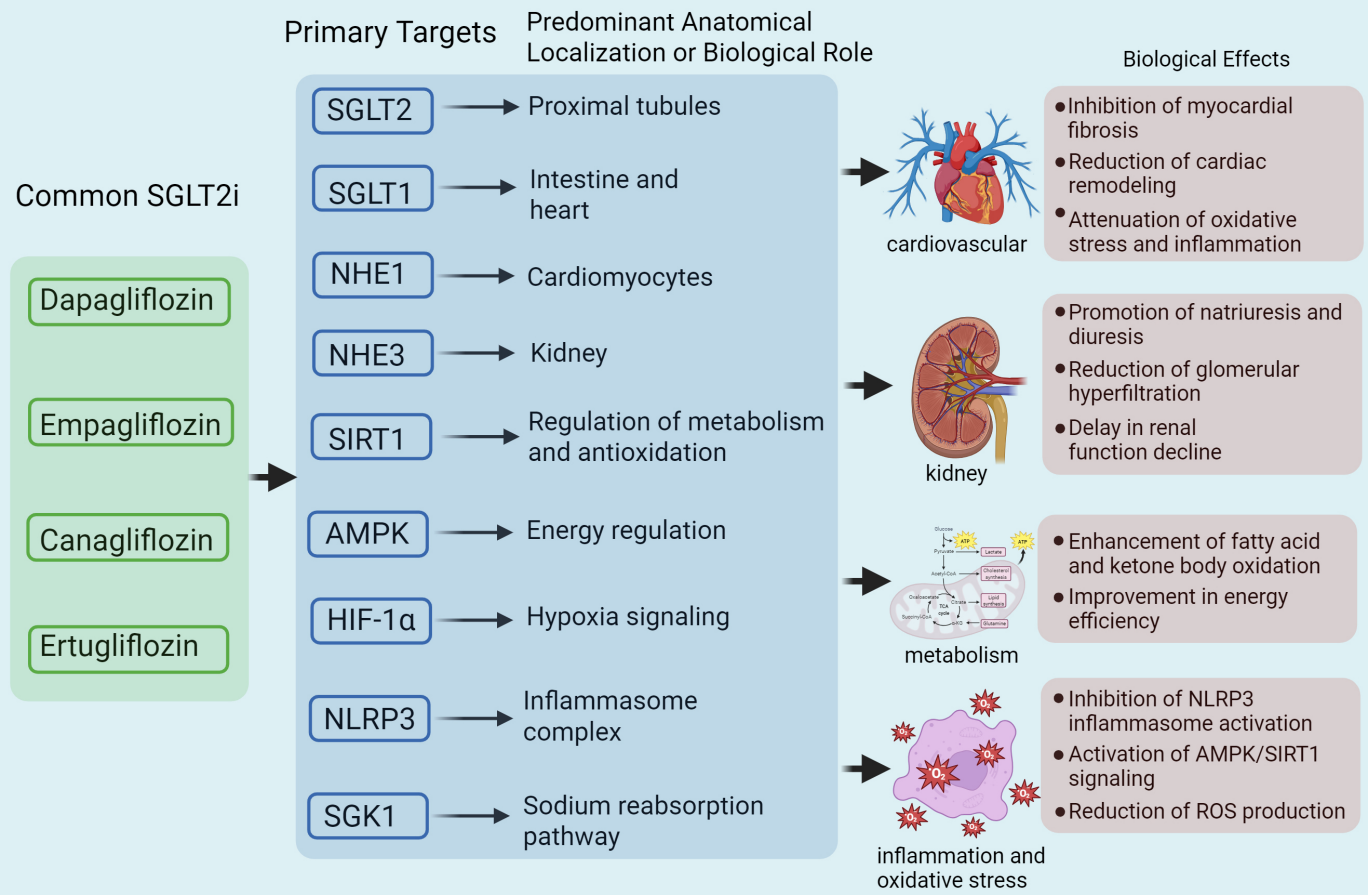
**Mechanisms by which SGLT2is treat HF through different targets**. Dapagliflozin, empagliflozin, canagliflozin, and ertugliflozin exert various functional effects by targeting multiple molecular pathways, including SGLT2, sodium–glucose cotransporter 1 (SGLT1), sodium–hydrogen exchanger 1 (NHE1), NHE3, sirtuin 1 (SIRT1), AMP-activated protein kinase (AMPK), hypoxia-inducible factor 1-alpha (HIF-1α), NOD-like receptor protein 3 (NLRP3), and serum- and glucocorticoid-inducible kinase 1 (SGK1). The far-right layer of the network diagram illustrates the multisystem mechanisms of action of SGLT2is. ROS, reactive oxygen species. Fig. 2 was drawn using BioRender.

SGLT2is improve hemodynamics and reduce blood pressure by inducing osmotic diuresis, which decreases intravascular fluid volume [22] (Fig. 2). This reduction in blood pressure not only lowers the resistance to cardiac output (afterload) but also reduces venous return (preload), thereby alleviating the initial workload on the heart and enabling more efficient cardiac pumping [23]. Studies have reported a modest antihypertensive effect of SGLT2is, with an estimated average blood pressure reduction of 2.46/1.46 mm Hg [24]. Notably, this antihypertensive effect persists even in patients with impaired renal function, suggesting mechanisms beyond simple diuresis, including improved endothelial function, reduced arterial stiffness, and modulation of sympathetic nervous activity [25].

The reduction in preload is primarily achieved through diuretic and natriuretic effects, which decrease venous return [26]. Cardiac magnetic resonance imaging studies have demonstrated reduced end-diastolic volume following treatment with empagliflozin, consistent with decreased preload [27]. These changes collectively reduce cardiac workload, particularly in patients with HF. The DAPA-HF trial showed that dapagliflozin reduced the risk of the composite endpoint of cardiovascular death or worsening HF by 26% compared to placebo, suggesting that SGLT2is may directly enhance blood flow by optimizing cardiac loading conditions [28]. In addition, SGLT2is indirectly promote vasodilation by improving endothelial function, further contributing to improved hemodynamics [29]. While the primary mechanism involves renal-mediated volume regulation, emerging evidence also points to direct effects on cardiac cells, such as reductions in inflammation and oxidative stress [30].

Interstitial edema, characterized by fluid accumulation in tissue interstices, is common in HF patients [31]. SGLT2is mitigate interstitial edema by increasing urine output and sodium excretion, thereby reducing fluid retention [32]. Studies suggest that the diuretic effect of SGLT2is preferentially targets interstitial fluid rather than solely reducing plasma volume [33]. Mathematical modeling has shown that dapagliflozin has a lesser impact on plasma volume compared to the angiotensin-converting enzyme inhibitor (ACEI) enalapril, minimizing the risk of hypovolemia-induced reflex neurohumoral activation and electrolyte imbalances [34]. The RECEDE-CHF trial reported an increase in daily urine output of 545 mL with SGLT2is, of which 312 mL was electrolyte-free water clearance, contributing to the alleviation of fluid overload [35]. Although evidence on the long-term diuretic effects of SGLT2is remains inconsistent, these effects are considered beneficial in reducing pulmonary edema and improving congestive HF symptoms.

### 3.2 Regulation of Cardiac Metabolism

SGLT2is exert multifaceted regulatory effects on cardiac metabolism in HF, by coordinated modulation of glucose, energy, and lipid metabolism [36]. By inducing mild ketosis, SGLT2is influence glucose metabolism by promoting lipolysis and reducing glucose utilization, thereby enhancing ketone body production [37]. These ketone bodies serve as an efficient energy substrate for the heart, which is particularly beneficial in improving cardiac function in HF [38]. Studies suggest that SGLT2is reduce glucose uptake in cardiomyocytes by inhibiting glucose transporter 1 (GLUT1), which is beneficial in HF patients with impaired metabolic function [39].

In individuals with marked insulin resistance or poorly controlled type 2 diabetes, chronic suppression of glucose oxidation and elevated circulating free fatty acids create a highly inefficient metabolic environment [40]. Preclinical studies have shown that hearts from insulin-resistant models exhibit a greater shift toward ketone oxidation and mitochondrial coupling efficiency after SGLT2is exposure compared to non-diabetic controls [41]. Conversely, in non-diabetic or mildly insulin-resistant heart failure patients, baseline ketone oxidation is already relatively preserved, and the benefits of SGLT2is may derive more from systemic effects—such as improved renal—cardiac crosstalk, reduced inflammation, and enhanced autophagy—than solely from altering direct metabolic substrates [42]. Clinical metabolomic analyses from the DAPA-HF and EMPEROR studies have shown that although plasma ketone levels increase modestly in all patients, the correlation between increased ketones and clinical outcomes is strongest in those with higher baseline insulin resistance or hyperglycemia [43]. These findings support a model in which SGLT2is-mediated cardioprotection is multifactorial and context-dependent: the relative contribution of ketone metabolism predominates in metabolically impaired states, whereas hemodynamic and anti-inflammatory mechanisms may dominate in metabolically healthier individuals [42]. Recognizing this heterogeneity will be essential for tailoring therapeutic strategies and identifying patient subgroups that are most likely to benefit from SGLT2is-induced metabolic remodeling.

In terms of energy metabolism, SGLT2is enhances ATP production by promoting mitochondrial biogenesis and improving mitochondrial function [44]. They facilitate ketone body utilization, a metabolic pathway that is more efficient than fatty acid oxidation, thereby increasing the heart’s energy utilization efficiency [38]. Furthermore, SGLT2is appear to inhibit sodium–hydrogen exchanger 1 (NHE1), reducing intracellular sodium and calcium concentrations in cardiomyocytes [45]. This contributes to maintaining ion homeostasis and preventing calcium overload, which is critical for HF patients.

Regarding lipid metabolism, SGLT2is promote the oxidation of long-chain fatty acids, thereby reducing the accumulation of toxic lipid intermediates, a process particularly relevant in HF, where dysregulated lipid metabolism contributes to lipid accumulation and oxidative stress [46]. In addition, SGLT2is exert cardioprotective effects by modulating levels of lipid metabolism-related hormones, such as leptin and adiponectin [47].

### 3.3 Attenuation of Inflammatory Responses

Chronic inflammation is a hallmark of HF, and SGLT2is mitigate this inflammatory state, thereby reducing myocardial injury [48]. Interleukin-1β (IL-1β), a central mediator of the pro-inflammatory cascade, promotes cardiomyocyte apoptosis, endothelial dysfunction, and myocardial fibrosis [49]. SGLT2is suppresse the production of reactive oxygen species (ROS) and inhibits the activation of nuclear factor κB (NF-κB), directly blocking the assembly and activation of the NOD-like receptor protein 3 (NLRP3) inflammasome, which in turn reduces the maturation and release of IL-1β and IL-18 [50]. As a key transcription factor in inflammatory responses, NF-κB inhibition by SGLT2is lowers both systemic and cardiac-localized inflammation, enhancing cardiomyocyte survival and vascular endothelial function [25]. This effect is mediated through the activation of AMP-activated protein kinase (AMPK) and inhibition of inhibitor-kappaB kinase β (IKKβ) phosphorylation, which prevents NF-κB nuclear translocation and reduces the transcription of pro-inflammatory genes such as tumor necrosis factor-α (TNF-α), IL-6, and IL-8 [51].

Excessive activation of signal transducer and activator of transcription 3 (STAT3) is associated with myocardial hypertrophy and fibrosis, and its inhibition mitigates pathological ventricular remodeling [52]. SGLT2is reduce IL-6 levels, thereby inhibiting Janus kinase 2 (JAK2)/STAT3 phosphorylation and blocking pro-inflammatory and pro-fibrotic signaling pathways [53]. SGLT2is attenuate stimulation by interferon-γ (IFN-γ) and lipopolysaccharide (LPS), reducing the expression of M1 macrophage markers such as inducible nitric oxide synthase (iNOS) and CD86 [54]. By activating peroxisome proliferator-activated receptor γ (PPARγ) and AMPK signaling, SGLT2is enhance the expression of M2 macrophage markers, including arginase 1 (Arg1) and CD206, promoting anti-inflammatory and tissue repair mechanisms [55]. This shift in the M1/M2 macrophage balance toward M2 polarization reduces the release of pro-inflammatory cytokines (TNF-α, IL-1β) while increasing the production of anti-inflammatory cytokines (IL-10, TGF-β), thereby suppressing myocardial inflammation and fibrosis [56].

SGLT2is modulate immune responses by inhibiting the IL-6/STAT3 pathway, which reduces the generation of Th17 cells and promotes regulatory T cell (Treg) proliferation, thereby limiting excessive immune activation [57]. A decreased Th17/Treg ratio mitigates IL-17-mediated myocardial inflammation and fibrosis [58]. By alleviating hyperglycemia and oxidative stress, SGLT2is inhibit the release of neutrophil extracellular traps (NETs) by neutrophils, reducing microvascular embolism and inflammatory damage in the myocardium [59]. NETs are critical mediators of myocardial ischemia-reperfusion injury and HF progression, and their reduction helps protect the myocardial microcirculation [60].

### 3.4 Reduction of Oxidative Stress Injury

Oxidative stress plays a pivotal role in the progression of HF, contributing to endothelial dysfunction, cardiac remodeling, and impaired contractility [25]. SGLT2is mitigate oxidative stress injury through multiple mechanisms. In mouse models of HF, empagliflozin significantly reduced cardiac ROS production while upregulating the expression of endogenous antioxidants [61]. SGLT2is may modulate intracellular sodium homeostasis by inhibiting NHE1 and the sodium channel Nav1.5, thereby attenuating oxidative stress [45].

Autophagy, a critical process for clearing damaged cellular components, particularly ROS-generating dysfunctional mitochondria, is enhanced by SGLT2is [62]. Studies indicate that SGLT2is promote autophagy by activating AMPK, SIRT1, and hypoxia-inducible factor pathways, facilitating the removal of these deleterious components [39]. Given the close interplay between oxidative stress and inflammation, the anti-inflammatory effects of SGLT2is may indirectly reduce oxidative stress [50]. By suppressing pro-inflammatory cytokines and modulating immune cell activity, SGLT2is mitigate oxidative damage due to inflammation [63].

SGLT2is reduce oxidative stress by altering myocardial energy substrate utilization, shifting from carbohydrates to more efficient substrates such as fatty acids and ketone bodies [37]. This metabolic shift enhances mitochondrial function, lowers ROS production, and further alleviates oxidative stress by improving insulin sensitivity and glucose control [9]. SGLT2is demonstrate significant reductions in oxidative stress in non-diabetic HF rat models, suggesting that their protective effects may be independent of glycemic control [64]. This indicates that SGLT2is exert broader cardioprotective effects through direct cardiac actions and metabolic regulation, beyond mechanisms associated with diabetes. Furthermore, SGLT2is enhance the antioxidant capacity of cardiomyocytes by activating antioxidant enzyme systems, providing additional protection against injury from oxidative stress [65].

### 3.5 Regulation of the Neuroendocrine System

SGLT2is exert their therapeutic effects in HF through modulation of multiple neuroendocrine systems. The DAPA-HF trial evaluated the efficacy of dapagliflozin in HF, while the EMPEROR-Reduced and EMPEROR-Preserved trials investigated empagliflozin in HF patients with varying ejection fractions [66, 67, 68]. These trials demonstrated that SGLT2is significantly reduce cardiovascular mortality and HF hospitalization rates, suggesting a potential role in improving cardiac function through neuroendocrine regulation [7].

SGLT2is promote urinary excretion, reducing plasma sodium levels, which in turn lowers renin and aldosterone levels, thereby suppressing the activity of the renin-angiotensin-aldosterone system (RAAS) [32]. Studies have shown that SGLT2is, such as empagliflozin, significantly decrease plasma aldosterone levels in HF patients, contributing to reduced fluid retention and vasoconstriction [69]. Excessive RAAS activation in HF exacerbates fluid retention and vascular constriction, and its inhibition helps alleviate these pathological processes [70].

In HF, increased activation of the sympathetic nervous system (SNS) leads to increases in heart rate, myocardial contractility, and vascular resistance. SGLT2is mitigate SNS activity by improving fluid balance and metabolic control [71]. Recent studies have reported decreased variability in heart rate and reduced resting heart rate following dapagliflozin treatment, indicating enhanced autonomic nervous system function, likely through decreased sympathetic activity, which reduces heart rate and cardiac workload [72].

Beyond their effects on traditional RAAS and SNS pathways, SGLT2is modulate additional hormones, such as brain natriuretic peptide (BNP) and glucagon-like peptide-1 (GLP-1), offering novel insights into their broad therapeutic benefits. SGLT2is may lower BNP levels, which is a reflection of reduced cardiac stress [73]. GLP-1, a hormone with cardioprotective properties, enhances myocardial contractility and reduces apoptosis [74]. Dapagliflozin has been shown to increase GLP-1 levels, potentially providing additional protection by improving cardiac contractile function [6].

### 3.6 Suppression of Myocardial Fibrosis and Improvement of Ventricular Remodeling

Ventricular remodeling is a central pathological process in the progression of HF, characterized by hypertrophy of cardiomyocytes, apoptosis, and interstitial fibrosis, leading to ventricular dilation and contractile dysfunction [75]. SGLT2is reduce blood volume through osmotic diuresis (sodium and glucose excretion), lowering preload, while also decreasing afterload, thereby reducing cardiac workload [32]. This alleviation of cardiac stress decreases energy demands, improves myocardial oxygen supply-demand balance, and suppresses compensatory cardiomyocyte hypertrophy [72]. SGLT2is indirectly inhibit NHE1 activity in cardiomyocyte membranes, reducing intracellular sodium levels and subsequently attenuating sodium–calcium exchanger (NCX)-mediated calcium overload, which mitigates calcium-dependent contractile dysfunction and mitochondrial oxidative stress [45]. Increased phosphorylation of AMPK suppresses excessive mammalian target of rapamycin (mTOR) activation, restoring the balance between autophagy and apoptosis, thereby reducing cardiomyocyte apoptosis and necrosis [76]. SGLT2is induce mild ketosis (elevated β-hydroxybutyrate levels), promoting preferential myocardial utilization over free fatty acids, enhancing ATP production efficiency, and ameliorating deficits in myocardial energy metabolism [77]. This also reduces myocardial lipid accumulation and lipotoxicity-mediated endoplasmic reticulum stress, preserving mitochondrial function [78].

Myocardial fibrosis, a key contributor to increased ventricular stiffness and diastolic dysfunction, is modulated by SGLT2is through multiple mechanisms. SGLT2is downregulate transforming growth factor-beta (TGF-β) expression and inhibit Smad2/3 signaling, which attenuates fibroblast-to-myofibroblast transformation and collagen synthesis [79]. They upregulate matrix metalloproteinase (MMP) activity to promote collagen degradation while inhibiting tissue inhibitors of metalloproteinases (TIMPs), thereby reversing excessive extracellular matrix (ECM) deposition [80]. Preclinical models indicate that short-term SGLT2is administration (2–4 weeks) can rapidly attenuate profibrotic signaling by downregulating TGF-β1/Smad3 phosphorylation and suppressing inflammatory mediators such as IL-6 and TNF-α [81]. In diabetic and pressure-overload rodent models, empagliflozin and dapagliflozin reduced myocardial TGF-β expression and macrophage infiltration within 2–3 weeks of treatment, before significant changes in ECM content became apparent [82]. In contrast, long-term interventions (8–24 weeks in animal studies or ≥6 months in clinical trials) are associated with measurable reductions in collagen I/III deposition, normalization of MMP-2/MMP-9 activity, and improvements in myocardial stiffness and left ventricular mass [83]. These findings suggest that early anti-inflammatory and anti-oxidative signaling precedes—and may causally mediate—the subsequent structural reversal of fibrosis. Cardiac magnetic resonance (CMR) and echocardiographic studies have demonstrated improvements in extracellular volume fraction (ECV) and left ventricle (LV) mass index typically after 6–12 months of therapy, whereas circulating markers of fibrosis (e.g., procollagen type I C-terminal propeptide (PICP), galectin-3) may decline within the first 3 months [84]. By suppressing nicotinamide adenine dinucleotide phosphate (NADPH) oxidase activity and enhancing superoxide dismutase (SOD) expression, SGLT2is reduce ROS levels, limiting oxidative damage [63]. In addition, they decrease levels of TNF-α, IL-6, IL-1β, and monocyte chemoattractant protein-1 (MCP-1), inhibiting M1 macrophage polarization and localized myocardial inflammation [54]. Osmotic diuresis reduces activation of the RAAS, attenuating aldosterone-driven fibroblast proliferation and collagen synthesis [85].

The EMPEROR-Reduced trial demonstrated that SGLT2is significantly reduced left ventricular mass index (LVMI), which is indicative of the reversal of myocardial hypertrophy [8]. SGLT2is also limit ventricular dilation (reducing left ventricular end-systolic volume index (LVESVi)), restore sphericity index, and optimize contractile synchrony [27]. By reducing myocardial stiffness and the E/e^′^ ratio, SGLT2is improve left ventricular filling pressure, alleviating diastolic dysfunction [86]. The DAPA-HF and EMPEROR-Reduced trials confirmed that dapagliflozin and empagliflozin significantly lowered the risk of cardiovascular death and HFrEF, with subgroup analyses revealing improvements in left ventricular ejection fraction (LVEF) and reductions in N-terminal pro-B-type natriuretic peptide (NT-proBNP) [7, 87]. Cardiac magnetic resonance imaging studies have also shown that SGLT2is-treated groups exhibit reduced markers of myocardial fibrosis, such as ECV, and decreased myocardial fibrosis, and showed evidence of reverse ventricular remodeling [88].

## 4. Clinical Evidence and Therapeutic Potential of SGLT2is in HF

SGLT2is have demonstrated significant efficacy and broad therapeutic potential in the treatment of HFrEF, HFpEF, and HFmrEF [28]. Several clinical trials have confirmed that SGLT2is significantly reduce the risk of cardiovascular death and HF hospitalization across a broad range of ejection fractions [7] (Table 1, Ref. [7, 8, 10, 23, 35, 87, 89, 90, 91]). The DAPA-HF trial showed that dapagliflozin reduced the risk of cardiovascular death or HF hospitalization by 26% and all-cause mortality by 17% in HFrEF patients [87]. Similarly, the EMPEROR-Reduced trial demonstrated that empagliflozin reduced the risk of cardiovascular death or HF hospitalization by 25% in HFrEF patients, with benefits that were independent of the patient’s diabetes status [10]. The EMPEROR-Preserved trial was the first to show that empagliflozin reduced the risk of cardiovascular death or HF hospitalization by 21% in HFpEF patients, while the DELIVER trial reported an 18% reduction in the risk of the primary endpoint in HFpEF patients with an LVEF >40% [89]. Subgroup analyses indicate consistent benefits in patients with an LVEF of 40%–49%, with reductions in cardiovascular death or the risk of HF hospitalization ranging from 22% to 25%, consistent with outcomes observed in patients with HFrEF and HFpEF [92].

**Table 1.** **Summary of key clinical trials and core evidence for SGLT2is in HF**.

Trial name	HF classification	Intervention regimen	Key subgroup analysis findings	References
EMPEROR-Reduced	HFrEF	Empagliflozin vs. Placebo	Subgroup aged ≥75 years is consistent with younger patients	[7, 10]
EMPEROR-Preserved	HFpEF	Empagliflozin vs. Placebo	Consistent benefits across LVEF 50%–59% and LVEF ≥60% subgroups.	[8, 89]
DEFINE-HF Trial	HFrEF	Dapagliflozin vs. Placebo	No significant difference in RRR between T2DM patients and non-T2DM patients; Benefit retained in eGFR 25–45 mL/min/1.73 m^2^ subgroup	[23]
RECEDE-CHF	HFrEF/HFpEF	Dapagliflozin vs. Placebo	No significant subgroup differences; No increased risk of acute kidney injury observed	[35]
DELIVER	HFpEF/HFmrEF	Dapagliflozin vs. Placebo	Patients with atrial fibrillation, no difference from those without atrial fibrillation	[89, 90]
DAPA-HF	HFrEF/HFpEF/HFmrEF	SGLT2is Group vs. Conventional Therapy Group (RAASis + β-blockers ± MRAs)	Subgroup aged ≥75 years is consistent with younger patients	[87, 91]

HFrEF, HF with reduced ejection fraction; HFpEF, HF with preserved ejection fraction; HFmrEF, HF with mid-range ejection fraction; LVEF, left ventricular ejection fraction; T2DM, type 2 diabetes mellitus; eGFR, estimated glomerular filtration rate; RAASis, Renin–Angiotensin–Aldosterone System inhibitors; MRAs, Mineralocorticoid Receptor Antagonists; RRR, Relative Risk Reduction.

SGLT2is also exhibit significant advantages in special high-risk populations, such as those with comorbid chronic kidney disease (CKD). Both dapagliflozin and empagliflozin significantly reduce the risk of cardiovascular death or HF hospitalization while slowing the progression of CKD [93]. Although SGLT2is are generally considered safe and well-tolerated, it is important to highlight the risk of euglycemic diabetic ketoacidosis (eDKA), a rare but potentially life-threatening complication. However, emerging evidence suggests that this condition can occasionally occur in non-diabetic heart failure patients receiving SGLT2is, particularly during catabolic or increased stress conditions such as prolonged fasting, infection, perioperative periods, or excessive alcohol consumption [94]. In patients with advanced CKD, the risk–benefit balance requires particular attention. Reduced renal clearance may alter the handling of glucose and ketone, and CKD-related malnutrition or infection could predispose patients to ketosis under stress [95]. Therefore, clinicians should educate patients and caregivers to recognize early symptoms of ketoacidosis (nausea, vomiting, abdominal pain, dyspnea) and advise temporary discontinuation of SGLT2is during acute illness, surgery, or reduced oral intake [96]. In patients with acute HF or worsening HF, SGLT2is, when used in combination therapy, increase urine output without compromising renal function [97]. SGLT2is also demonstrate synergistic effects when combined with conventional HF medications, further optimizing therapeutic outcomes [98].

The safety profile of SGLT2is in HF treatment has been extensively validated. Multiple large-scale randomized controlled trials (RCTs) and meta-analyses have confirmed that SGLT2is significantly reduce the risk of cardiovascular death and hospitalization across various HF phenotypes without introducing additional safety concerns [90, 99, 100]. Pooled analyses from the DAPA-HF and EMPEROR-Reduced trials demonstrated that dapagliflozin and empagliflozin significantly lowered mortality across the full range of ejection fractions, with adverse event rates comparable to placebo [7]. Real-world studies, such as the CVD-REAL study, further corroborate the safety of SGLT2is in HF management, showing high patient adherence and tolerability with low rates of adverse events [101].

Despite the significant advancements achieved with SGLT2is in the management of HF, further in-depth studies are needed to elucidate their molecular mechanisms and cellular signaling pathways to develop more effective therapeutic strategies and optimize drug combinations. Future research should prioritize large-scale, long-term RCTs to rigorously evaluate the efficacy and safety of SGLT2is across diverse patient populations, with a particular emphasis on HFpEF and specific subgroups. These subgroups include, but are not limited to, patients with LVEF >60%, elderly patients (aged ≥75 years), those with advanced CKD, and individuals with comorbidities such as obesity, atrial fibrillation, or pulmonary hypertension [91]. The burden of HF is particularly higher in older adults, yet elderly patients (≥75 years) remain underrepresented in major RCTs. Subgroup analyses from landmark trials have nonetheless provided consistent evidence that the efficacy of SGLT2is extends to this population. In DAPA-HF, approximately 24% of participants were aged ≥75 years, and the relative risk reduction for the composite endpoint of cardiovascular death or worsening HF was similar to that in younger cohorts [102]. The EMPEROR-Reduced and EMPEROR-Preserved trials demonstrated no attenuation of treatment benefit with increasing age, confirming that SGLT2is’ cardioprotective effects are age-independent [103]. In the various trials, the incidence of key adverse events—such as volume depletion, genital infections, and decline in renal function—was only modestly increased in older participants, with no significant increase in hypoglycemia or ketoacidosis [104]. SGLT2is initiation was well tolerated even in patients with advanced age and moderate CKD, supporting their feasibility in geriatric practice when appropriately monitored. Future trials should investigate the efficacy of SGLT2is in underrepresented populations, such as those with genetic predispositions to HF or non-Caucasian ethnic groups, to ensure the broad applicability of these findings.

Optimizing the combination of SGLT2is with other HF therapies, including RAAS inhibitors, β-blockers, MRAs, glucagon-like peptide-1 receptor agonists (GLP-1RAs), and angiotensin receptor-neprilysin inhibitors (ARNIs), is critical for achieving therapeutic synergy and minimizing adverse effects [105]. SGLT2is promote mild osmotic diuresis and natriuresis, which may potentiate the volume-depleting effects of loop diuretics. In the DAPA-HF and EMPEROR trials, a modest increase in volume-related adverse events (e.g., hypotension, dehydration) was observed when high-dose loop diuretics were co-administered [102]. Accordingly, clinicians should consider gradual uptitration or a temporary reduction in the dose of loop diuretics when initiating SGLT2is, particularly in euvolemic or frail patients. No clinically significant pharmacokinetic interactions have been reported between SGLT2is and ARNIs. However, both classes can reduce blood pressure and promote natriuresis, which may increase the risk of symptomatic hypotension, especially in elderly or CKD patients [95]. Careful monitoring of blood pressure and renal function during the early phase of combination therapy is recommended. No major pharmacokinetic interactions are known, and evidence suggests additive benefits on cardiac remodeling and reductions in mortality [84]. Nonetheless, caution is warranted when SGLT2is are introduced in patients with borderline renal function or those at risk for hyperkalemia, as concurrent MRAs therapy may amplify these effects.

Personalized treatment strategies tailored to individual patient characteristics—such as comorbidities (e.g., T2DM, CKD, or coronary artery disease), renal function (e.g., eGFR levels), baseline LVEF, and frailty status—are essential to enhance treatment efficacy and safety. Natriuretic peptides (BNP, NT-proBNP) are well-established indicators of ventricular stress and may identify patients most likely to experience rapid hemodynamic improvement with SGLT2is [106]. Early reductions in NT-proBNP after initiation have been correlated with improved outcomes in both HFrEF and HFpEF populations [107]. Estimated glomerular filtration rate (eGFR) and albuminuria are key determinants of both efficacy and tolerability [108]. Clinical data suggest that SGLT2is retain cardiorenal protective effects as low as eGFR thresholds of 20–25 mL/min/1.73 m^2^, but initial eGFR decline and long-term slope stabilization vary by baseline renal status [109]. Parameters such as HbA1c, fasting ketone levels, and high-sensitivity C-reactive protein (hsCRP) may provide additional insights into metabolic adaptation and residual inflammatory risk during therapy [110].

A major paradigm shift introduced by large-scale randomized trials was the observation that the cardiovascular and renal benefits of SGLT2is extend beyond glucose lowering. Subgroup analyses from pivotal trials—including DAPA-HF, EMPEROR-Reduced, and EMPEROR-Preserved—have consistently shown that the relative risk reduction in heart failure hospitalization and cardiovascular death with SGLT2is therapy is comparable in patients with and without T2DM [76]. In trial DAPA-HF trial, for example, dapagliflozin reduced the composite outcome of worsening HF or cardiovascular death by 26% in patients without T2DM, closely matching the 25% reduction observed in those with T2DM [106]. Similar findings were reported in the EMPEROR-Reduced and DELIVER trials, underscoring that SGLT2is confer cardioprotective effects independent of glycemic status [103, 109]. However, subtle distinctions do exist between diabetic and non-diabetic HF populations. Patients with T2DM tend to have a higher baseline risk of renal dysfunction, volume overload, and microvascular disease, which may increase the absolute risk reduction observed with SGLT2is [107]. Conversely, non-diabetic HF patients may exhibit greater improvements in metabolic efficiency and myocardial energetics, albeit with slightly increased vigilance required for the development of volume depletion or euglycemic ketoacidosis [96]. Long-term safety monitoring of SGLT2is in non-diabetic HF patients is particularly important, especially for potential risks such as diabetic ketoacidosis, urogenital infections, and volume depletion [18]. Expanded real-world evidence studies and post-marketing surveillance are needed to assess these risks over extended periods and in varied clinical contexts. Furthermore, future research should clarify the optimal timing and sequencing of SGLT2is initiation relative to other HF therapies, particularly in patients with acute HF or recent decompensation [100]. Through these comprehensive research efforts, SGLT2is have the potential to further transform the landscape of HF management, offering improved prognosis and quality of life for a broader patient population.

## 5. Conclusions

SGLT2is exhibit remarkable efficacy and considerable potential in the management of HF. These agents exert their therapeutic effects through multiple mechanisms, including optimization of hemodynamics, regulation of cardiac metabolism, attenuation of inflammatory responses and oxidative stress, modulation of neuroendocrine pathways, and suppression of myocardial fibrosis. SGLT2is demonstrate robust performance in special populations and when used in combination with other HF therapies. Their safety has been consistently validated across numerous studies, with low rates of adverse events. Future research should prioritize elucidating the molecular mechanisms of SGLT2is, conducting additional large-scale clinical trials, exploring optimal combination therapies, developing personalized treatment strategies, and monitoring long-term safety in non-diabetic HF patients. With these advancements, SGLT2is are poised to play an increasingly pivotal role in the treatment of HF, thereby enhancing patient prognosis and quality of life.
